# A Simple Competing Endogenous RNA Network Identifies Novel mRNA, miRNA, and lncRNA Markers in Human Cholangiocarcinoma

**DOI:** 10.1155/2019/3526407

**Published:** 2019-03-24

**Authors:** Cheng Zhang, Chunlin Ge

**Affiliations:** Department of Pancreatic and Biliary Surgery, The First Hospital of China Medical University, Shenyang, Liaoning 110001, China

## Abstract

**Background:**

Cholangiocarcinoma (CCA) is the second most common malignant primary liver tumor and has shown an alarming increase in incidence over the last two decades. However, the mechanisms behind tumorigenesis and progression remain insufficient. The present study aimed to uncover the underlying regulatory mechanism on CCA and find novel biomarkers for the disease prognosis.

**Method:**

The RNA-sequencing (RNA-seq) datasets of lncRNAs, miRNAs, and mRNAs in CCA as well as relevant clinical information were obtained from the Cancer Genome Atlas (TCGA) database. After pretreatment, differentially expressed RNAs (DERNAs) were identified and further interrogated for their correlations with clinical information. Prognostic RNAs were selected using univariate Cox regression. Then, a ceRNA network was constructed based on these RNAs.

**Results:**

We identified a total of five prognostic DEmiRNAs, 63 DElncRNAs, and 90 DEmRNAs between CCA and matched normal tissues. Integrating the relationship between the different types of RNAs, an lncRNA-miRNA-mRNA network was established and included 28 molecules and 47 interactions. Screened prognostic RNAs involved in the ceRNA network included 3 miRNAs (hsa-mir-1295b, hsa-mir-33b, and hsa-mir-6715a), 7 lncRNAs (ENSG00000271133, ENSG00000233834, ENSG00000276791, ENSG00000241155, COL18A1-AS1, ENSG00000274737, and ENSG00000235052), and 18 mRNAs (ANO9, FUT4, MLLT3, ABCA3, FSCN2, GRID2IP, NCK2, MACC1, SLC35E4, ST14, SH2D3A, MOB3B, ACTL10, RAB36, ATP1B3, MST1R, SEMA6A, and SEL1L3).

**Conclusions:**

Our study identified novel prognostic makers and predicted a previously unknown ceRNA regulatory network in CCA and may provide novel insight into a further understanding of lncRNA-mediated ceRNA regulatory mechanisms in CCA.

## 1. Introduction

Cholangiocarcinoma (CCA) is an epithelial cell malignancy arising from different locations within the biliary tree. According to its anatomical location, CCA is classified into intrahepatic (iCCA), perihilar (pCCA), and distal (dCCA) subtypes [1]. Surgical treatment is the preferred option for all subtypes [2]; however, most patients with CCA present with unresectable or metastatic disease. As one of the deadliest cancers, patient prognoses remain poor after systemic chemotherapy [3]. More than 90% of patients die within five years, and the majority of patients survive less than 12 months after diagnosis [4]. Additionally, the incidence of CCA seems to be increasing in many western countries [2], and because of this increase, cumulative CCA mortality rates have also increased by 39% [5]. The increased incidence and mortality have both contributed to the rising interest in this cancer.

In 2011, Leonardo Salmena and colleagues first proposed the famous competitive endogenous RNA (ceRNA) hypothesis, positing that RNAs can engage in crosstalk with one another and manipulate biological functions independently of protein translation [7]. lncRNAs are endogenous noncoding RNAs longer than 200 nucleotides and constitute the largest portion of the human noncoding transcriptome [8]. Recent studies have revealed that lncRNAs can act as ceRNAs to compete with mRNAs for binding to miRNAs like sponges, and thereby regulate the expression level of genes [9, 10]. Moreover, this pattern has been extensively demonstrated to play important roles in carcinogenesis and in the development of numerous cancers, including bladder cancer, thyroid cancer, and gastric cancer [11–13]. However, compared with other malignancies, the ceRNA research in CCA remains sparse. Sun et al. discovered that KCNQ1OT1 can act as a ceRNA to improve CCA progression by regulating the miR-140-5p/SOX4 axis [14]. In addition, lncRNA H19, HULC, and TUG1 have also been shown to promote CCA progression as ceRNAs [15, 16]. However, the essential biomarkers and mechanisms for the development and progression of CCA remain unclear.

With the rapid development of gene profile and next generation sequencing technology, bioinformatics analysis can provide more valuable information for new research [17]. However, like most bioinformatics studies on CCA, Huang and Zhong's groups have only identified differentially expressed genes (DEGs) between CCA and normal tissues and constructed a protein–protein interaction (PPI) network [18, 19]. An analysis of lncRNA and miRNA functions and their interactions is absent. Additionally, bioinformatics studies related to the ceRNA network in CCA are rare. At present, only a few articles [20–22] can be found. In contrast to all the aforementioned studies, all of the molecules that we selected in the construction of our network were proven to be differentially expressed and prognosis-related. In addition, we determined the relationships between RNAs from the expression data in the CCA samples rather than in RNA interaction databases. The method of functional enrichment analysis was also different. We built a GO terms network to further visualize the biological process and molecular function of crucial genes. Then, the KEGG, Reactome and WikiPathways were used to enrich the related mRNAs pathways as opposed to the sole use of KEGG, which can help to obtain a more comprehensive analytic result. Therefore, there is some merit to our research in terms of a deeper understanding of CCA.

In the present study, with the aim of identifying the potential ceRNA interactions that contribute to patient survival, we constructed a ceRNA regulatory network that included mRNAs that were dysregulated in CCA as compared to normal tissues. The expression and clinical data were acquired from the Cancer Genome Atlas (TCGA), a publicly funded project that aims to catalogue and discover major cancer-causing genomic alterations to create a comprehensive “atlas” of cancer genomic profiles [23]. All RNAs participating in the construction of the network are demonstrated to be dysregulated and prognosis-related. Because of this strict standard, the network appears to be simple; however, compared with networks containing hundreds of molecules, it may provide more reliable clues for ongoing basic research.

## 2. Materials and Methods

### 2.1. Acquisition of Clinical Characteristics and RNA-Seq Data

The clinical and mRNA expression data of 48 CCA patients were obtained from the TCGA database (https://portal.gdc.cancer.gov/). Exclusion criteria were set as follows: (i) histologic diagnosis excluding CCA; (ii) incomplete RNA expression data for analysis; and (iii) overall survival time less than 30 days. Ultimately, 33 patients were included in survival analysis. The clinical and pathological characteristics of these patients are summarized in Table 1. Then, lncRNA, miRNA, and mRNA expression files that included 36 CCA samples and nine adjacent normal samples were also downloaded from the TCGA portal.

### 2.2. Identification of Differentially Expressed RNAs

RNAs with average expression values greater than 1 were considered for further analysis. lncRNAs and mRNAs are defined based on the annotations of the GeneCards database (https://www.genecards.org/). We used the R and Bioconductor packages of DESeq2 and limma to explore the significantly differentially expressed mRNAs (DEmRNAs), lncRNAs (DElncRNAs), and miRNAs (DEmiRNAs) between CCA tissues and normal tissues. Fold-change was used to measure the change degree. For p-values, the false discovery rate (FDR) was applied to determine the significance threshold for multiple tests. The cut-off value was |log_2_FoldChange| > 2 for mRNA and lncRNA, |log_2_FoldChange| > 1.5 for miRNA. FDR < 0.05 was considered significant.

### 2.3. Survival Analysis

Univariate Cox regression was performed to evaluate the association between survival and the expression levels of DEmRNAs, DElncRNAs and DEmiRNAs. Then, an intermediate expression value was used as the cut-off point and the patients were divided into high-expression and low-expression groups. Survival curves were generated using the Kaplan-Meier method, and the log-rank test was used to compare the differences between groups. P < 0.05 was the threshold for significance. DEmRNAs, DElncRNAs, and DEmiRNAs related to survival were considered as prognostic DEmRNAs, prognostic DElncRNAs, and prognostic DEmiRNAs, respectively.

### 2.4. Construction of the ceRNA Network

The interactions between prognostic DEmiRNAs and DElncRNAs and between prognostic DEmiRNAs and DElncRNAs were evaluated by Pearson's correlation. lncRNAs can compete with mRNAs for binding to miRNAs like sponges to regulate posttranscriptional regulation; therefore, the relationship between these RNAs is negative. Pearson's correlation coefficient <-0.5 was considered as significant. Cytoscape (version 3.6.1; http://www.cytoscape.org/) software was utilized to draw this network.

### 2.5. Functional Enrichment Analysis

Generally, lncRNAs and miRNAs do not encode proteins but their functions are associated with related protein coding genes. To understand the underlying biological mechanisms, Gene Ontology (GO) annotation and pathway analyses of prognostic DEmRNAs were conducted by DAVID (https://david.ncifcrf.gov/), Kyoto Encyclopedia of Genes and Genomes (KEGG, https://www.kegg.jp/), Reactome (https://reactome.org/), and WikiPathways (https://www.wikipathways.org/). GO terms and pathway analyses with P<0.05 were considered to be significantly enriched functional annotations. The relationships between GO terms and related genes were visualized by Cytoscape.

## 3. Results

### 3.1. Identification of DElncRNAs, DEmRNAs, and DEmiRNAs in CCA Patients

**Figure 1:**
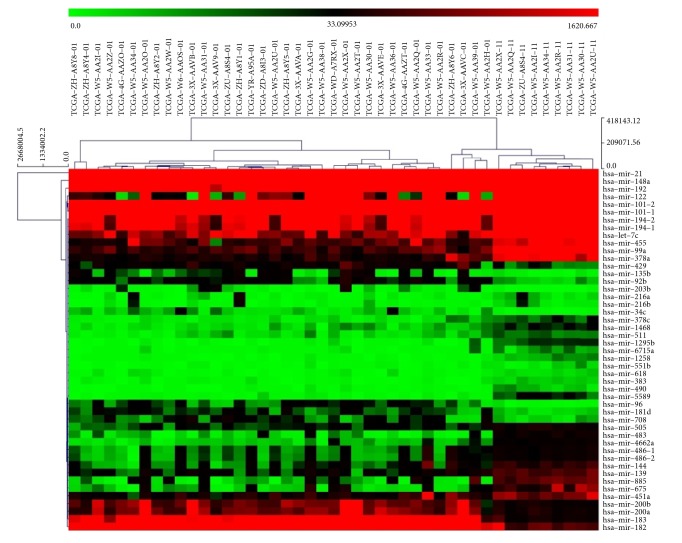
Expressions of differentially expressed miRNAs in different samples. X-axis represents samples, Y-axis represents miRNAs, red stands for upregulation, and green stands for downregulation [6].

**Figure 2:**
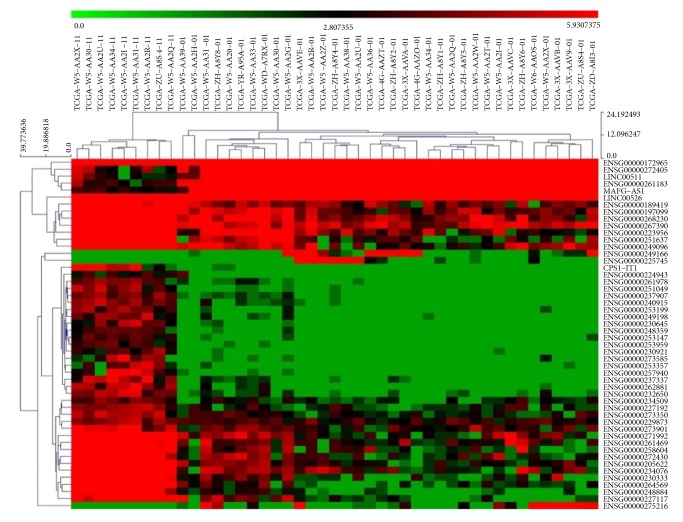
Expressions of differentially expressed top 50 lncRNAs in different samples. X-axis represents samples, Y-axis represents lncRNAs, red stands for upregulation, and green stands for downregulation [6].

**Figure 3:**
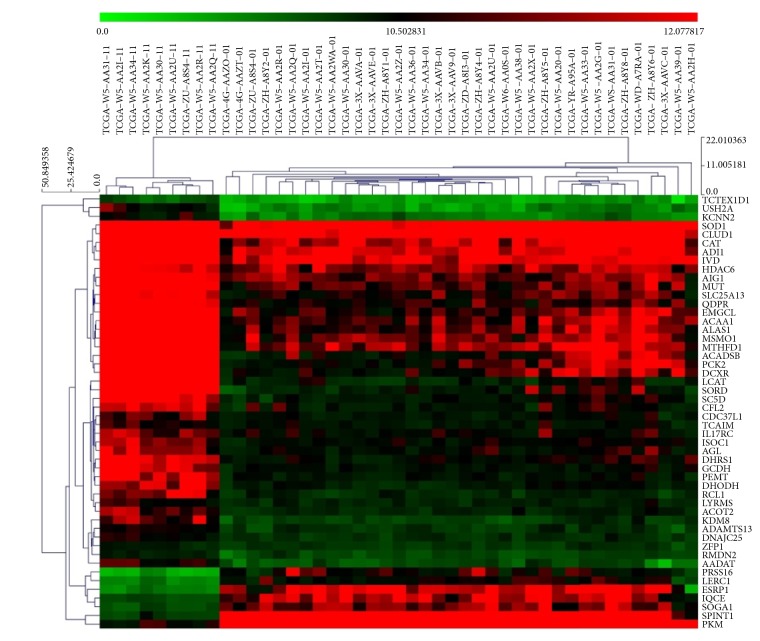
Expressions of top 50 differentially expressed mRNAs in different samples. X-axis represents samples, Y-axis represents mRNAs, red stands for upregulation, and green stands for downregulation [6].

The lncRNA, mRNA, and miRNA expression data of 36 CCA samples and 9 adjacent normal samples were downloaded from the TCGA project. After annotating different RNA types, we identified a total of 7783 lncRNAs, 17685 protein coding mRNAs, and 370 miRNAs with average expression values greater than 1. Under the criteria of differential analysis, as described above, 3657 DEmRNAs (2287 upregulated and 1370 downregulated), 1622 DElncRNAs (970 upregulated and 652 downregulated), and 66 DEmiRNAs (23 upregulated and 43 downregulated) were significantly dysregulated. Heat maps of the clustering analysis of these RNAs are presented in Figures 1–3 and show that the three kinds of RNAs can well-distinguish tumor tissues from normal tissues.

### 3.2. Survival Analysis of Dysregulated Genes

**Figure 4:**
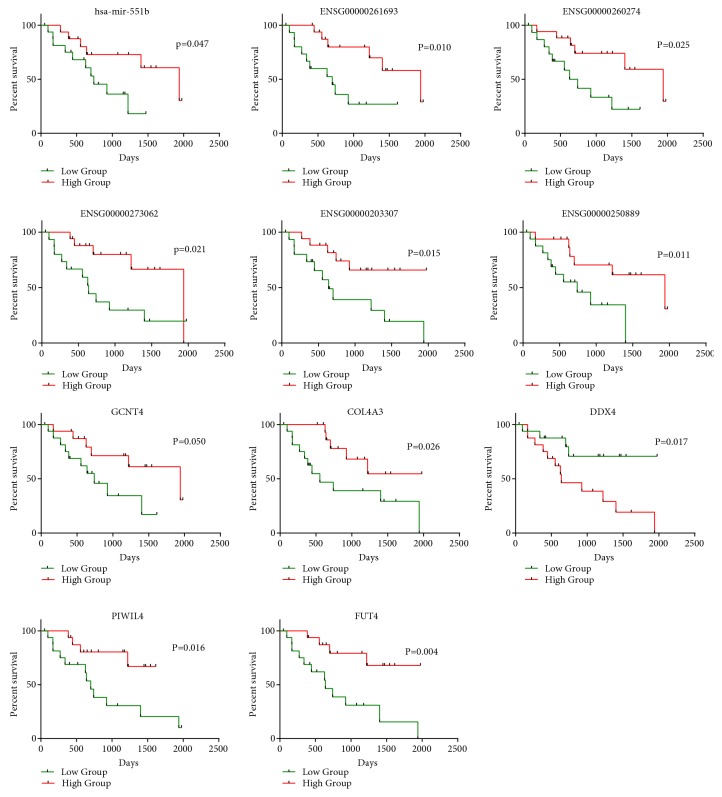
Kaplan-Meier curve analysis of DEmiRNAs, DElncRNAs, and DElncRNAs for the overall survival in CCA patients.

Univariate Cox regression analysis was performed to examine the association between the expression of the three kinds of dysregulated RNAs and the OS of patients with CCA. As shown in Tables 2–4, five miRNAs (hsa-mir-1266, hsa-mir-1295b, hsa-mir-33b, hsa-mir-551b, and hsa-mir-6715a); 63 lncRNAs including ENSG00000236117, ENSG00000249856, HOTAIR, and AGAP2-AS1; and 90 mRNAs including GCNT4, PYGB, DDX4, and PIWIL4 were identified to be prognostic DEmRNAs, prognostic DElncRNAs, and prognostic DEmiRNAs, respectively. An intermediate expression value of these prognostic RNAs was used as the cut-off point, the patients were divided into high-expression and low-expression groups, and KM survival analysis was performed. We found a significant survival difference between two groups (Figure 4).

### 3.3. Functional Enrichment Analysis of Prognostic DEmRNAs

**Figure 5:**
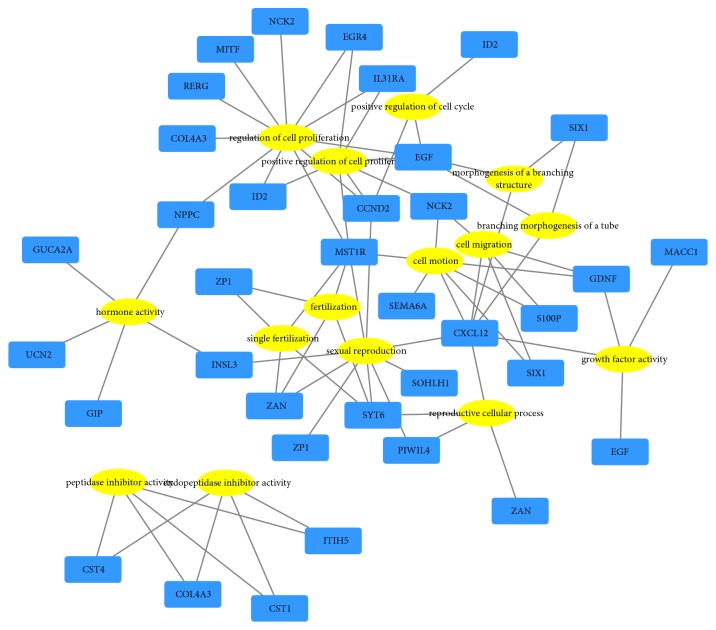
Gene ontology (GO) network of prognostic DEmRNAs. Yellow stands for GO terms and blue stands for mRNAs.

After performing prognostic DEmRNAs gene ontology analysis (GO) with DAVID, we found that these genes were classified into the following three functional groups: the molecular function group, the biological process group, and the cellular component group. As shown in Table 5, in the biological process group, genes were mainly enriched in the regulation of cell proliferation, cell motion, cell migration, and reproductive cellular process. In the molecular function group, genes were mainly enriched in hormone activity, endopeptidase inhibitor activity, and growth factor activity. In the cellular component group, genes were mainly enriched in extracellular region. The results are further visualized by a network (Figure 5). Next, pathway enrichment was performed using KEGG, Reactome, and WikiPathways. We found that target genes were associated with salivary secretion, prolactin signaling, and complexed PIWIL4 binding to cleaved transposon RNA (Table 6).

### 3.4. Construction of the ceRNA Network

**Figure 6:**
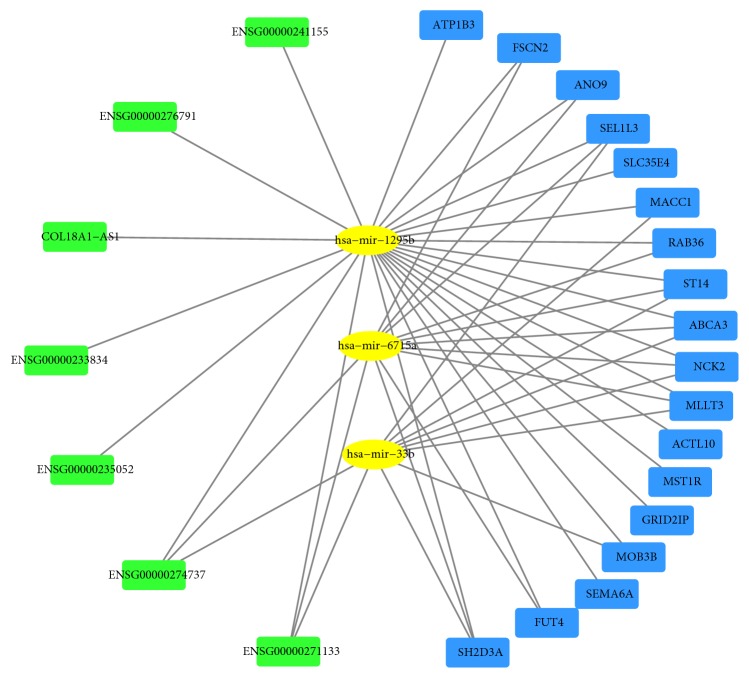
CeRNA regulatory network. Yellow represents miRNAs, blue represents mRNAs, and green represents lncRNAs.

First, we acquired prognostic DEmiRNAs and DElncRNAs interactions based on Pearson's correlation test. Three miRNAs (hsa-mir-1295b, hsa-mir-33b, and hsa-mir-6715a) and seven lncRNAs (ENSG00000271133, ENSG00000233834, ENSG00000276791, ENSG00000241155, COL18A1-AS1, ENSG00000274737, and ENSG00000235052) were demonstrated to be correlative. Then, we acquired 18 related mRNAs including ANO9, FUT4, MLLT3, and ABCA3 using the three abovementioned miRNAs. Finally, combining the relationships between different types of RNAs, an integrated lncRNA-miRNA-mRNA network was established and consisted of 28 molecules and 47 interactions (Figure 6).

## 4. Discussion

Cholangiocarcinoma (CCA), which emerges from the malignant proliferation of cholangiocytes, is an epithelial tumor of the biliary trees [24]. Concealed symptoms, a lack of early diagnostic markers, and low sensitivity to conventional radiotherapy and chemotherapy treatments are the crucial causes underlying poor prognoses for CCA patients [25]. Due to high morbidity and mortality in CCA, revealing the underlying molecular mechanisms, identifying molecular biomarkers for early diagnosis, prevention, and personalized therapy are critically important and highly demanded.

Currently, increasing studies have demonstrated the crucial biological functions of ceRNAs involved in the development and progression of many cancers. Additionally, the ceRNA hypothesis suggests a novel regulatory mechanism that can be mediated by lncRNAs. For example, the lncRNA XIST promotes the epithelial to mesenchymal transition of retinoblastoma via sponging miR-101 [26]. Moreover, Dong et al. found that knockdown of the lncRNA HOXA-AS2 could suppress chemoresistance in acute myeloid leukemia via the miR-520c-3p/S100A4 axis [27]. With the deeper understanding of ceRNA network, we can no longer regard miRNAs, lncRNAs, or mRNAs as separate elements in disease. Following the expression change of the affected miRNAs, the expression of target genes regulated by miRNAs will also change, leading to the occurrence of a variety of diseases, including tumors. Besides, the mechanism of occurrence and progression of cancer is too complex and the effect of single gene or single pathway is very limited. Therefore, systematic construction and analysis of ceRNA network can provide us with a more specific area to research and a new perspective on the underlying mechanism of cancer. However, few studies have described the interactions between different types of RNAs in CCA, and ceRNA network research has been rarely reported.

In our study, we chose three prognosis-related miRNAs (hsa-mir-1295b, hsa-mir-33b, and hsa-mir-6715a) to build the ceRNA network. hsa-mir-33b has been reported to be involved in multiple types of human cancers. For example, Tian et al. reported that hsa-mir-33b is downregulated in hepatocellular carcinoma and suppresses the proliferation and metastasis of HCC cells through the inhibition of Sal-like protein 4 (SALL4) [28]. Moreover, hsa-mir-33b can inhibit lung adenocarcinoma cell growth, invasion, and epithelial-mesenchymal transition by suppressing Wnt/*β*-catenin/ZEB1 signaling [29]. hsa-mir-33b has also been shown to be dysregulated and prognosis-related in ovarian cancer [30]. However, a report of hsa-mir-33b has not been made for CCA, and the underlying regulatory mechanisms of this important molecule remain largely unclear. In addition, hsa-mir-1295b was found to be significantly downregulated in colorectal cancer [31, 32], and to the best of our knowledge, no research on hsa-mir-1295b has been reported for any other type of cancer. Regarding hsa-mir-6715a, we could not identify any report related to cancer. Therefore, it is necessary to further research these miRNAs. Our study demonstrates that low-expression levels of hsa-mir-33b, hsa-mir-1295b, and hsa-mir-6715a are associated with poor OS in CCA patients, and these miRNAs are candidate novel therapeutic targets that require further fundamental research.

Many dysregulated lncRNAs could be involved in tumor evolution and progression. In our ceRNA network, seven lncRNAs (ENSG00000271133, ENSG00000233834, ENSG00000276791, ENSG00000241155, COL18A1-AS1, ENSG00000274737, and ENSG00000235052) were identified. Although COL18A1-AS1 has been reported to act as a biomarker involved competitive endogenous RNA in clear cell renal cell carcinoma [33, 34], little is known about the function of COL18A1-AS1 in CCA, and research on other lncRNAs in CCA is scarce. Therefore, further investigations are needed to clarify the functions of these lncRNAs in CCA and other cancers.

In the present study, 90 prognostic DEmRNAs were identified based on univariate Cox regression analysis. GO enrichment and pathway analyses were performed to further study the biological functions of these genes. It was found that these genes were mainly involved in regulating cell proliferation, cell motion, cell migration, and reproductive cellular process via GO terms. The pathway analysis showed significant enrichment in prolactin signaling, RA biosynthesis signaling, PIWI-interacting RNA (piRNA) biogenesis, and IL-2 signaling, among others. These GO terms and pathways are all crucial for the development and progression of cancer [35–37]. Further studies are required, and our prediction of these functional mechanisms may provide a foundation for future research.

After systematically analyzing the expression data in CCA tissues and normal tissues using TCGA, we identified prognostic tumor-specific miRNAs, lncRNAs, and mRNAs and constructed a ceRNA network with these genes. This network can prompt us to explore the regulation of ceRNAs in CCA further. For example, metastasis-associated in colon cancer 1 (MACC1), identified in colon cancer patients for the first time, has been found to play multiple important roles in many malignancies, such as hepatocellular cancer and lung cancer [38–40]. Lederer et al. found that MACC1 expression in hilar cholangiocarcinoma tissue was significantly higher than in corresponding normal tissue. They also discovered that patients with high MACC1 had a significantly shorter overall and disease-free survival [41]. However, despite the important clinical significance, the underlying mechanism of MACC1 remains largely unknown. In our network, MACC1 was also proved to be prognosis-related and we obtained a meaningful lncRNA-miRNA-mRNA regulatory axis involved MACC1 (COL18A1-AS1–hsa-mir-1295b–MACC1). The prediction of this axis can provide us with a clue that COL18A1-AS1 and hsa-mir-1295b may participate in the protumor process of MACC1 and these molecules may become novel diagnostic biomarkers and therapeutic targets for CCA. Researchers can carry out further fundamental studies to validate this axis. Consequently, through this study, we can not only obtain important lncRNAs, miRNAs, and mRNAs related to the occurrence and progression of CCA but also further understand its underlying mechanism, which will contribute to the early diagnosis, treatment, and prognosis of CCA.

Although several similar studies have been performed, our research still provides some novel insights. Wan et al. [20] constructed a ceRNA network based on DEmRNAs, DElncRNAs, and all miRNAs. miRNAs involved in the network did not prove to be differentially expressed. Moreover, in contrast to all other ceRNA network bioinformatic studies [20–22], to the best of our knowledge, all the RNA molecules that we selected in the construction of our network were differentially expressed and prognosis-related. In addition, we acquired the interactions between RNAs from the expression profiles of CCA tissues rather than RNA interaction databases. The relationship between different types of RNAs was confirmed to be accordant with their expression levels in CCA samples, which adds credibility to our network. We also built a GO terms network to further visualize the biological process and molecular functions of crucial genes. The KEGG, Reactome, and WikiPathways databases, rather than the KEGG database alone, were used to enrich the related mRNA pathways, which confers our study with more comprehensive analytic results. Finally, under our strict standard, we constructed a simple network containing fewer RNAs than other studies. However, compared with networks including hundreds of molecules, our network may provide a more reliable and precise basis for further fundamental research.

There are several limitations to our study. First, we only acquired 36 tumor samples and nine nontumor samples. This small sample number may introduce false positives and reduce the reliability of our findings. Second, all of our samples and clinical data were based on TCGA. Another independent database that includes large-scale multicenter clinical samples should be used to verify our findings. Third, a lack of biological validation is present in our study. Our future research will focus on validating these RNAs in CCA and uncovering the molecular mechanisms of ceRNAs* in vivo* and* in vitro *to validate our discovery.

In conclusion, we identified prognostic tumor-specific miRNAs, lncRNAs, and mRNAs and constructed a ceRNA network with these genes. Our study predicted a previously unknown ceRNA regulatory network and may provide novel insights into and a better understanding of the development and progression of CCA.

## Figures and Tables

**Table 1:** Clinicopathological characteristics of 33 CCA patients.

Characteristic	Subtype	No. of cases	overall survival (Days)
Age (years)	< 65	16(48.5%)	942.4
	≥65	17(51.5%)	729.2
Gender	Male	14(42.4%)	695.5
	Female	19(57.6%)	932.8
Pathologic stage	Stage I	18(54.5%)	904.9
	Stage II	8(24.2%)	686.9
	Stage III	1(3.0%)	1077.0
	Stage IV	6(18.2%)	768.8
Pathologic T	T1	18(54.5%)	904.9
	T2	10(30.3%)	669.0
	T3	5(15.2%)	899.0
Pathologic N	NO	25(75.8%)	920.8
	N1	5(15.2%)	642.2
	NX	3(9.1%)	414.7
Pathologic M	M0	27(81.8%)	861.6
	M1	4(12.1%)	826.0
	MX	2(6.1%)	453.0
Vital status	Alive	17(51.5%)	991.1
	Dead	16(48.5%)	664.0

**Table 2:** DEmiRNAs related to overall survival time.

Terms	HR[exp(coef)]	coef	95% CI	95% CI	Z	P value
			lower	upper		
hsa-mir-6715a	1.17154675	0.158325	0.049902	0.266747	2.862051	0.004209
hsa-mir-33b	1.18342871	0.168416	0.028973	0.307858	2.367207	0.017923
hsa-mir-1266	1.12023629	0.11354	0.015197	0.211882	2.262838	0.023646
hsa-mir-551b	0.34086099	-1.07628	-2.10373	-0.04883	-2.05312	0.040061
hsa-mir-1295b	1.12448821	0.117328	0.001963	0.232693	1.993315	0.046227

**Table 3:** DElncRNAs related with overall survival time.

Terms	HR[exp(coef)]	coef	95% CI	95% CI	Z	P value
			lower	upper		
ENSG00000250889	0.528519	-0.63768	-1.0061	-0.26925	-3.39236	0.000693
ENSG00000273062	0.378218	-0.97229	-1.5422	-0.40237	-3.34375	0.000827
ENSG00000261693	0.398936	-0.91895	-1.52033	-0.31758	-2.99499	0.002745
ENSG00000260274	0.372464	-0.98762	-1.68543	-0.2898	-2.77392	0.005539
ENSG00000203307	0.483861	-0.72596	-1.24584	-0.20607	-2.73684	0.006203
ENSG00000254486	2.552133	0.936929	0.26283	1.611029	2.724149	0.006447
ENSG00000231680	0.478653	-0.73678	-1.27574	-0.19782	-2.67935	0.007377
ENSG00000257894	0.565936	-0.56927	-0.98686	-0.15169	-2.67191	0.007542
ENSG00000250362	2.461851	0.900914	0.237324	1.564503	2.66092	0.007793
ENSG00000227014	0.494181	-0.70485	-1.23045	-0.17925	-2.6284	0.008579
ENSG00000261318	0.468954	-0.75725	-1.32498	-0.18952	-2.61424	0.008943
ENSG00000236117	1.505228	0.408944	0.099647	0.718242	2.591407	0.009558
ENSG00000272622	2.006874	0.696578	0.167071	1.226086	2.578375	0.009927
ENSG00000249856	0.647258	-0.43501	-0.76886	-0.10116	-2.55382	0.010655
HOTAIR	1.30067	0.26288	0.058595	0.467165	2.522134	0.011665
AGAP2-AS1	2.149615	0.765289	0.160601	1.369976	2.480518	0.013119
COL18A1-AS1	0.590817	-0.52625	-0.94272	-0.10978	-2.47659	0.013264
ENSG00000276791	0.505092	-0.68302	-1.22693	-0.1391	-2.4612	0.013847
ENSG00000278214	0.54384	-0.6091	-1.09835	-0.11985	-2.4401	0.014683
ENSG00000275216	1.237912	0.213426	0.041969	0.384883	2.439721	0.014699
ENSG00000227619	1.43819	0.363385	0.069165	0.657606	2.420708	0.01549
ENSG00000233491	1.51341	0.414365	0.077512	0.751218	2.410966	0.01591
ENSG00000267629	1.93407	0.659626	0.114538	1.204715	2.371805	0.017701
ENSG00000270035	0.710753	-0.34143	-0.63079	-0.05207	-2.31264	0.020742
ENSG00000233834	0.664373	-0.40891	-0.75862	-0.05921	-2.29179	0.021918
ENSG00000260541	0.388057	-0.9466	-1.75669	-0.13651	-2.29025	0.022007
ENSG00000242147	1.441509	0.36569	0.048191	0.68319	2.257456	0.02398
ENSG00000271133	0.663591	-0.41009	-0.76665	-0.05353	-2.25421	0.024183
ENSG00000267108	0.652321	-0.42722	-0.79983	-0.05461	-2.24722	0.024626
ENSG00000232613	0.666056	-0.40638	-0.76118	-0.05158	-2.24489	0.024776

**Table 4:** DEmRNAs related to overall survival time.

Terms	HR[exp(coef)]	coef	95% CI	95% CI	Z	P value
			lower	upper		
GCNT4	0.554979	-0.58882	-0.90178	-0.27587	-3.68762	0.000226
PYGB	3.514451	1.256883	0.436048	2.077719	3.001145	0.00269
DDX4	2.168734	0.774144	0.235005	1.313283	2.814289	0.004889
PIWIL4	0.577875	-0.5484	-0.93535	-0.16145	-2.77772	0.005474
COL4A3	0.67404	-0.39447	-0.67317	-0.11577	-2.77409	0.005536
ANO9	0.614971	-0.48618	-0.83227	-0.14009	-2.75335	0.005899
INSL3	0.397032	-0.92374	-1.60152	-0.24596	-2.67122	0.007558
FUT4	0.463237	-0.76952	-1.33545	-0.20359	-2.66504	0.007698
HSPB9	0.310703	-1.16892	-2.02991	-0.30792	-2.66092	0.007793
C16orf96	0.519788	-0.65433	-1.13788	-0.17079	-2.65224	0.007996
CST1	1.273687	0.241916	0.063042	0.420789	2.650737	0.008032
GOLGA7B	0.638863	-0.44806	-0.77957	-0.11656	-2.64909	0.008071
GAD1	1.401288	0.337392	0.084864	0.58992	2.618623	0.008829
MLLT3	0.694936	-0.36394	-0.63733	-0.09054	-2.60902	0.00908
TECTB	1.63835	0.49369	0.120831	0.866549	2.59512	0.009456
RTBDN	1.481896	0.393323	0.095854	0.690791	2.591526	0.009555
CYP26A1	0.693564	-0.36591	-0.64397	-0.08785	-2.57922	0.009902
ABCA3	0.601324	-0.50862	-0.89573	-0.12151	-2.57519	0.010018
CXCL12	0.610734	-0.49309	-0.86845	-0.11774	-2.57476	0.010031
GOLGA8M	0.603553	-0.50492	-0.8904	-0.11945	-2.5673	0.01025
SOHLH1	2.198451	0.787753	0.175524	1.399981	2.521881	0.011673
FSCN2	0.453714	-0.79029	-1.40804	-0.17254	-2.5074	0.012162
GRID2IP	0.58603	-0.53438	-0.95221	-0.11655	-2.5067	0.012187
PRICKLE1	0.675506	-0.39229	-0.70756	-0.07702	-2.4388	0.014736
LGALS9B	1.364549	0.310824	0.060412	0.561236	2.432808	0.014982
UCN2	1.548267	0.437136	0.079748	0.794524	2.397312	0.016516
NCK2	0.484531	-0.72457	-1.32048	-0.12867	-2.38317	0.017164
GDNF	1.354636	0.303533	0.053253	0.553812	2.376996	0.017454
GUCA2A	0.828229	-0.18847	-0.34398	-0.03295	-2.37525	0.017537
MACC1	0.747344	-0.29123	-0.53412	-0.04834	-2.35001	0.018773

**Table 5:** GO enrichment analysis of prognostic DEmRNAs.

Category	Terms	Count	Genes	P Value
BP_FAT	GO:0019953~sexual reproduction	9	INSL3, ZP1, CCND2, ZAN, SYT6, PIWIL4, MST1R, SOHLH1, CXCL12	0.001362
BP_FAT	GO:0007338~single fertilization	4	ZP1, ZAN, SYT6, MST1R	0.002852
BP_FAT	GO:0042127~regulation of cell proliferation	11	RERG, NCK2, COL4A3, ID2, EGR4, CCND2, MITF, NPPC, MST1R, EGF, IL31RA	0.003643
BP_FAT	GO:0009566~fertilization	4	ZP1, ZAN, SYT6, MST1R	0.006184
BP_FAT	GO:0008284~positive regulation of cell proliferation	7	NCK2, ID2, EGR4, CCND2, MST1R, EGF, IL31RA	0.013322
BP_FAT	GO:0006928~cell motion	7	NCK2, SEMA6A, S100P, SIX1, MST1R, GDNF, CXCL12	0.024462
BP_FAT	GO:0045787~positive regulation of cell cycle	3	ID2, CCND2, EGF	0.029763
BP_FAT	GO:0048754~branching morphogenesis of a tube	3	SIX1, EGF, CXCL12	0.037869
BP_FAT	GO:0048610~reproductive cellular process	4	ZAN, SYT6, PIWIL4, CXCL12	0.041301
BP_FAT	GO:0016477~cell migration	5	NCK2, S100P, SIX1, GDNF, CXCL12	0.041515
BP_FAT	GO:0001763~morphogenesis of a branching structure	3	SIX1, EGF, CXCL12	0.047869
CC_FAT	GO:0005576~extracellular region	18	INSL3, COL4A3, ZP1, GIP, ADAMTS13, CST1, CXCL12, GDNF, RTBDN, UCN2, CST4, ST14, NPPC, KLK12, ITIH5, GUCA2A, EGF, TECTB	0.015299
CC_FAT	GO:0000267~cell fraction	12	PNPLA7, INSL3, SOAT2, ACY1, GIP, KLK12, GRID2IP, CYP26A1, FUT4, ALOX5, EGF, ABCA3	0.015849
CC_FAT	GO:0005625~soluble fraction	6	INSL3, ACY1, GIP, KLK12, ALOX5, EGF	0.018847
MF_FAT	GO:0005179~hormone activity	5	INSL3, UCN2, GIP, NPPC, GUCA2A	0.001642
MF_FAT	GO:0004866~endopeptidase inhibitor activity	4	COL4A3, CST4, ITIH5, CST1	0.030672
MF_FAT	GO:0030414~peptidase inhibitor activity	4	COL4A3, CST4, ITIH5, CST1	0.035142
MF_FAT	GO:0008083~growth factor activity	4	EGF, GDNF, CXCL12, MACC1	0.039932

**Table 6:** Pathway analysis of prognostic DEmRNAs.

GO ID	GO Term	Associated Genes Found	P Value
KEGG:04970	Salivary secretion	ATP1B3, CALML3, CST1, CST4	0.000790
R-HSA:5601883	Complexed PIWIL4 binds cleaved transposon RNA	DDX4, PIWIL4	0.001352
KEGG:04917	Prolactin signaling pathway	CCND2, CISH, ELF5	0.004151
R-HSA:5365859	RA biosynthesis pathway	CYP26A1, DHRS3	0.004593
R-HSA:5601884	PIWI-interacting RNA (piRNA) biogenesis	DDX4, PIWIL4	0.007906
R-HSA:419037	NCAM1 interactions	COL4A3, GDNF	0.016134
WP:49	IL-2 Signaling Pathway	CCND2, CISH	0.016134
R-HSA:5362517	Signaling by Retinoic Acid	CYP26A1, DHRS3	0.016871
R-HSA:1236394	Signaling by ERBB4	CXCL12, EGF	0.017623
WP:716	Vitamin A and Carotenoid Metabolism	CYP26A1, DHRS3	0.017623

## Data Availability

The data used to support the findings of this study are included within the article.
